# Densities of CO_2_‑Loaded and Unloaded 3‑Amino-1-propanol Aqueous Solutions and Their Blends with 2‑Amino-2-methyl-1-propanol at High Pressures

**DOI:** 10.1021/acsomega.5c07053

**Published:** 2025-10-24

**Authors:** Luana C. dos Santos, Eduardo Pérez, Alejandro Moreau, María D. Bermejo, José J. Segovia

**Affiliations:** † Foodomics Laboratory, Instituto de Investigación en Ciencias de la Alimentación (CIAL, CSIC-UAM), Nicolás Cabrera 9, Campus de Cantoblanco, Madrid 28049, Spain; ‡ Physical Chemistry Department, Universidad Complutense de Madrid, Madrid 28040, Spain; § BioEcoUva Research Institute on Bioeconomy, TERMOCAL-Thermodynamics and Calibration, 16782 University of Valladolid, Valladolid 47011, Spain; ∥ BioEcoUva Research Institute on Bioeconomy, PressTech, Department of Chemical Engineering and Environmental Technology, Universidad de Valladolid, Valladolid 47011, Spain

## Abstract

Carbon capture and storage and carbon capture and utilization are key technologies to reduce CO_2_ emissions by capturing and storing (or converting) CO_2_. In this context, amine-based aqueous solutions play a key role in these processes, especially because of their efficiency in chemically binding CO_2_. However, some physical properties under high pressure and temperature systems remain poorly reported in physical chemical databases. This work presents experimental data on the density of aqueous amine solutions of 3-amino-1-propanol (AP) when they are CO_2_-loaded and unloaded and its blends with 2-amino-2-methyl-1-propanol (AMP) (unloaded) under high-pressure conditions (up to 100 MPa) and at a wide temperature range (293.15–393.15 K). Density measurements were performed using a vibrating tube densimeter (Anton Paar DMA HPM), and data were correlated with a modified Tammann–Tait equation, resulting in excellent correlation. These results served as the support information for estimation of molar volumes and isothermal expansion coefficients. Overall, density increased with pressure and decreased with temperature for all amine solutions tested. At low AP concentrations, a local minimum was observed for the isothermal expansion coefficient, which is probably attributed to anomalous water compressibility. Additionally, the CO_2_ loading led to an increase in density and a decrease in thermal expansion coefficients. Finally, elemental analysis revealed a possible corrosion, especially in blends of AP + AMP and CO_2_-loaded solutions, providing valuable insights for material selection and process design.

## Graphical abstract

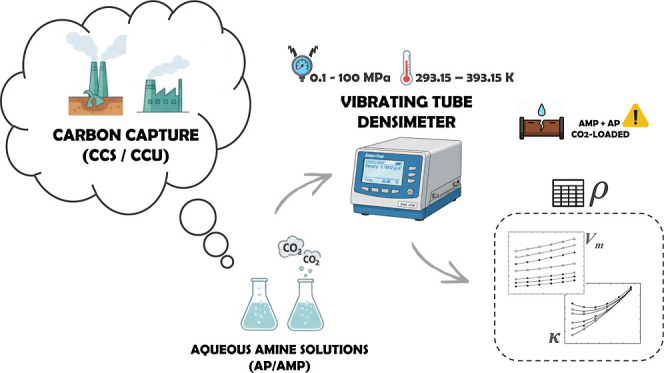

## 1 Introduction

In order to mitigate climate change issues, carbon capture and storage (CCS) and carbon capture and utilization (CCU) strategies are being quickly developed. The aim is mainly focused on developing new technologies to capture carbon dioxide (CO_2_) from industrial processes or directly from the atmosphere, transforming them into valuable products, rather than storing it underground.^1^ In this context, alternative processes have been studied, such as the capture of CO_2_ under high pressure and its in situ transformation into formic acid.^2^

Aqueous amine solutions have been extensively studied as a potential medium to capture CO_2_.^3,4^ Typically, suitable amines for CO_2_ absorption are those containing one or more alkyl alcohol groups.^5^ In general, 1 mol of tertiary amine is required for 1 mol of CO_2_ loading.^6^ In some cases, the beneficial properties of individual amines have been combined, as demonstrated by researchers who used blends of amines to study their efficiency in capturing CO_2_.^4^

Despite their efficiency, in order to design and scale up those processes, their physical properties (such as density, molar volume, and expansion coefficient) need to be addressed for proper process design (e.g., selection of appropriate vessel and pipeline materials and dimensions), and there is limited information in the literature, mostly when related to high pressure process. In addition, most of the reported studies in literature focus on how single amine solvents affect corrosion rates, and much less information is available on blended amine solvents, lacking details on their absorption mechanisms and their corrosion impact.^7^

Previously, our research group determined the physical properties of several aqueous amines, including diethanolamine (DEA), dimethylaminoethanol (DMAE), triethanolamine (TEA), and mixtures of piperazine (PZ) with DMAE.^8–10^ These amines are of particular interest for CO_2_ scrubbing under high-pressure conditions. More recently, Pérez-Milian^11^ et al. from the same research group reported density measurements over a wide range of temperatures, pressures, and amine mass fractions relevant to industrial CO_2_ capture. Their results showed that increasing the temperature leads to a rise in the isobaric heat capacity, with this effect being more pronounced at higher amine mass fractions.

2-Amino-2-methyl-1-propanol (AMP) and 3-Amino-1-propanol (AP) have been recently described as potential candidates for CO_2_ capturing, with similar efficiency as conventional processes,^12,13^ in which AMP performance is enhanced when they are mixed with other amines. For instance, Choi et al.^14^ have investigated the CO_2_ capture performance of amine mixtures of methylethanolamine (MEA) + AMP absorbents, under different mass fractions. The authors stated that adding MEA to AMP solutions enhances CO_2_ loading by 51.2% in comparison to a solution composed of 30 wt % of MEA. Despite MEA being the amine with higher investigation concerning CO_2_ capture, other amines are also tested, including the alkanolamine AP. AP contains a hydroxyl group, which helps proton transfer and consequently enhances the CO_2_ absorption rates. On the other hand, Hoff et al.^15^ describes blends of AMP + AP as effective absorbents, providing industrial evidence that both amines are considered useful together.

Therefore, this work introduces density results of loaded and unloaded amine solutions that still remained unknown. Accurate densities’ measurements of CO_2_ loaded and unloaded AP aqueous solutions and their blends with AMP at high pressures are presented. Moreover, calculations of respective molar volumes and thermal expansion coefficient were also presented.

## 2 Material and Methods

### 2.1 Materials

All chemicals used in this work were of high purity or analytical grade and are listed in Table 1, and no further purifying procedures were performed.

**Table 1:** Chemicals Used for Aqueous Amines Solutions Applied in This Work

chemical name	CAS #	mass fraction purity^a^	supplier
water (H_2_O)	7732-18-5	Conductivity ≤2 × 10^–6^ Ω^–1^·cm^–1^	Sigma-Aldrich
carbon dioxide (CO_2_)	124-38-9	≥99.9%	Linde
3-aamino-1-propanol (AP)	156-87-6	≥98.5%	Sigma-Aldrich
2-amino-2-methyl-1-propanol (AMP)	124-68-5	≥95.0%	Sigma-Aldrich

a According to supplier.

### 2.2 Thermophysical Properties of Aqueous Amines Solutions for CO_2_ Capture Applications

The aqueous solutions of AP and AMP were prepared by weighting according to the mass compositions (*w*) of 5, 10, 20, 30 and 40% of amine for binary system solutions (AP + H_2_O) and to the mass ratios AP/AMP of 1:2, 1:1, and 2:1 (*w* = 30%) when working with a tertiary solution. The samples were arranged using an analytical balance (Radwag scale model PS750/C/2) with a resolution of 1 mg. The amine mass fraction’s estimated expanded uncertainty (*k* = 2) is 0.0002. Pure components (AP, AMP, or H_2_O) were degassed previously to each density measurement or CO_2_-loading experiment (described in Section 2.3.2) in an ultrasonic bath (Branson 3200) during 40 min to avoid microbubbles in the pipeline that could affect thermodynamic properties of the system during the density measurements.

### 2.3 Density Measurements

#### 2.3.1 CO_2_ Unloaded Aqueous Amines

Amine solutions were submitted to density in a vibrant-tube density meter Anton Paar DMA HPM previously calibrated with water and vacuum as described in Segovia et al.^16^ A periodic checking of the calibration is performed with toluene to confirm deviations remain lower than the uncertainty of the measurements. The density meter is coupled with other substantial equipment, such as a thermostatic bath (Julabo HE F25), an evaluation unit mPDS 2000v3 to measure the vibrant period, an automatic pressure-controlled system besides a Pt100 thermoresistance temperature sensor calibrated with an expanded uncertainty (*k* = 2) of 0.02 K and pressure transducers (Druck DPI 104) with an expanded uncertainty (*k* = 2) of 0.02 MPa for the automated system.^16^ Before experiments, all the pipelines were rinsed with distilled water at least three times, and vacuum was applied until pressure was stable under 1 × 10^–3^ mbar. In sequential order, the liquid sample was filled into the system through a separating funnel, and the period (τ) values were obtained under the pressure range of 0.1–100 MPa when temperature was 293.15–353.15 K and 1–100 MPa when temperature applied was 373.15 and 393.15 K to avoid effects of water vapor pressure.

The uncertainty calculation was carried out following the procedure described by Segovia et al.^16^ and according to the document JCGM 100:2008.^17^ Uncertainty analysis showed an expanded relative uncertainty better than 0.1% for a 95.5% level of confidence.

#### 2.3.2 CO_2_-Loaded Aqueous Amine Solutions

AP solutions of *w* = 0.3 were submitted to CO_2_ loadings (α) of 0.2, 0.4, and 0.6 mol CO_2_ mol amine ^–1^. A 400 mL stainless steel reactor (Figure 1) was filled with approximately 150 mL of a degassed amine solution and closed. Following that, a CO_2_ stream (6 MPa, 298.15 K) pressurized the system up to 2 ± 0.5 MPa. Once the 2 MPa was reached, the CO_2_ flow was stopped and a pressure drop was observed through the pressure gauge. This process was repeated at least three times or until pressure drop was no longer detected, indicating that the CO_2_ capture by the amine solution reached its saturation.

**Figure 1:**
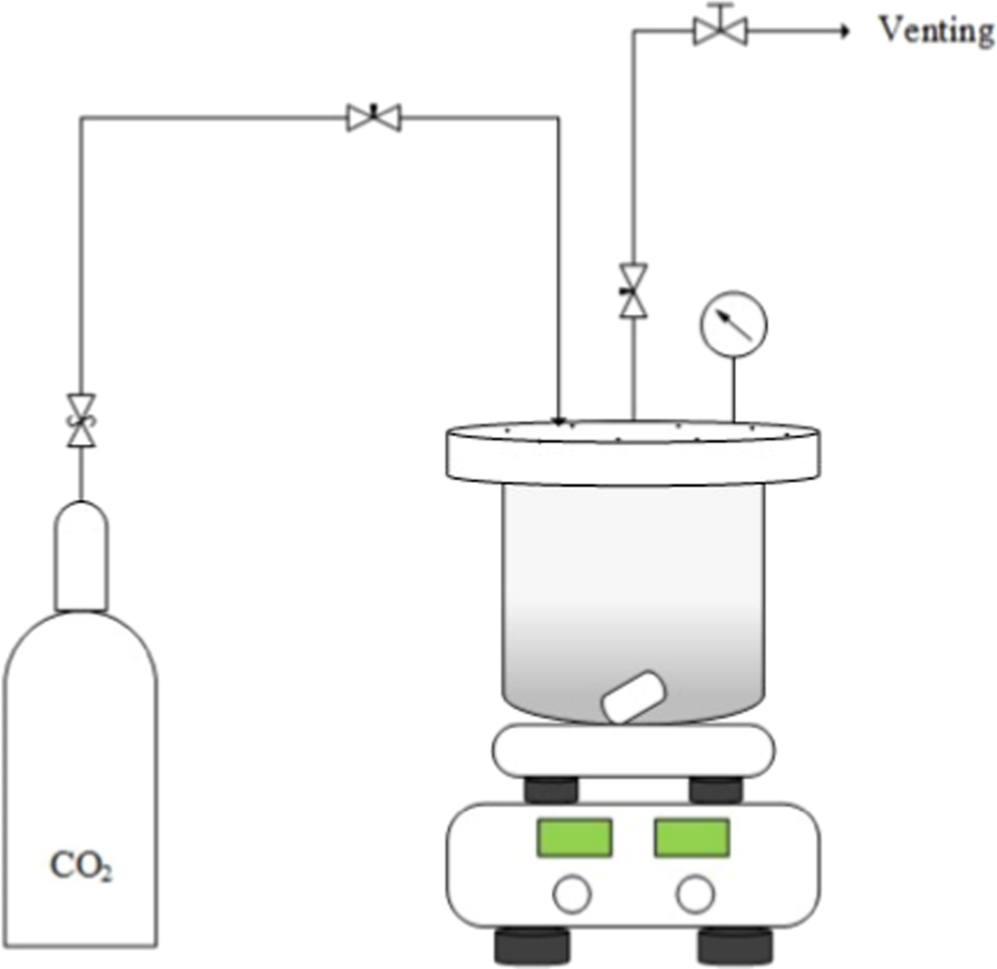
CO_2_-loading system for aqueous amine solutions.

The pH of the solutions was checked with a benchtop pH meter from Jenway (model 3505) coupled with an electrode (model 5021). CO_2_ concentration was measured using a Total Inorganic Carbon method implemented in a Total Organic Carbon Analyzer (TOC-V CHS) from Shimadzu with a repeatability of 1.5% in the CO_2_ content. This value has been taken as the standard deviation (*k* = 1) for the α (mol CO_2_ mol amine^–1^) uncertainty calculation and following the recommendations of the JCGM 100:2008 Guide.^17^ As a result, relative expanded uncertainty for the CO_2_ composition (*α*) of loaded aqueous amine solutions are better than 3% for a 95.5% level of confidence. For this analysis, a 500 ppm standard solution was prepared with a mixture of NaHCO_3_ and Na_2_CO_3_ (the calibration curve ranged from 0 to 500 ppm). The loaded aqueous amine solutions were diluted 100 times in water prior to the measurements. After the CO_2_ concentrated amine solution was characterized, it was used to prepare all the subsequent diluted loaded solutions which were also checked in terms of CO_2_ concentration following the same procedure previously described. The solutions were kept in glass flasks with N_2_ and kept in refrigeration and dark environments to avoid amine oxidation until density measurements.

Considering the uncertainty of the loaded aqueous solutions, density uncertainties have been recalculated for those systems. In Table 2, the density uncertainty budget for loaded aqueous amine solutions is presented.

**Table 2:** Uncertainty Budget for the Density Using the JCGM Guide^17^ for the Loaded Aqueous Amines Solutions

	units	estimated	divisor	*u*(*x*)/kg m^–3^	*u*(*x*)^2^
repeatability *u*(τ)	μs	5 × 10^–4^	1	7.5 × 10–3	5.65 × 10^–5^
resolution *u*(τ)		1 × 10^–3^	2√3	0.006	
reference material *u*(ρ_ref_)	kg m^–3^	0.01	√3		3.6 × 10^–5^
*u*(*A*(*T*))	kg m^–3^ μs^–2^	7 × 10^–8^	2	0.25	6.25 × 10^–2^
*u*(*B*(*T*,*p*))	kg m^–3^	0.5	2	0.25	6.25 × 10^–2^
calibration *u*(*T*)	K	0.02	2	0.014	1.96 × 10^–4^
resolution *u*(*T*)		0.01	2√3		
repeatability *u*(*T*)		5 × 10^–3^	1		
calibration *u*(*p*)	MPa	0.02	2	7.5 × 10^–3^	5.65 × 10^–5^
resolution *u*(*p*)		0.01	2√3		
repeatability *u*(*p*)		0.01	1		
alpha *u*(α)	mol CO_2_ mol amine^–1^	0.012	1	0.84	0.71
*u*(ρ)	kg m^–3^				0.91
			*U*(ρ)	(*k* = 2)	1.8
		(ρ = kg m^–3^)	988.1	0.20%	

### 2.4 Density Calculation and Data Fitting

The main goal of the vibrating tube densimeter is to achieve resonance with their natural frequency after the application of an electromagnetic field. Therefore, it is possible to obtain an oscillation period (*τ*) value every time the vibrating tube undergoes a change (*τ* is dependent on the total mass of the tube, i.e., every density change in the liquid filling will result in a different τ measurement). The period is then correlated to density through eq 1.

1 $$\rho =A\tau^{2}-B$$

where *ρ* is the liquid density inside the densimeter; *τ* is the oscillation period (time units), and *A* and *B* are the constants calculated after densimeter calibration with two fluids of well-known density within the desired range of *T* and *p*.

The experimental values were correlated using a modified Tammann–Tait empirical eq (eq 2) for each loaded and unloaded amines system.

2 $$\rho \left(T,p\right)=\frac{A_{0}+A_{1}T+A_{2}T^{2}}{1-C\ln\left(\frac{B_{0}+B_{1}T+B_{2}T^{2}+p}{B_{0}+B_{1}T+B_{2}T^{2}+p_{ref}}\right)}$$

where *A* is a function of temperature only; *B* is a function of *T* and *p*; *C* is dimensionless; *p*_ref_ is the reference pressure (0.1 MPa for the unloaded systems and 0.5 MPa for the loaded systems).

In order to statistically validate the fitting parameters, standard deviation (σ), maximum deviation (MD %), and average absolute deviation (AAD %) were calculated according to eqs 3–5.

3 $$\sigma =\sqrt{\left[\frac{1}{N-m}\right]\sum_{i=0}^{N}{\left(x_{\exp}-x_{calc}\right)}^{2}}$$

4 $$MD\;\left(\%\right)=\mathrm{Max}\cdot \left|\frac{x_{calc}-x_{\exp}}{x_{\exp}}\times 100\right|$$

5 $$AAD\;\left(\%\right)=\frac{\sum_{i=0}^{N}{\left(x_{\exp}-x_{calc}\right)}^{2}}{N}$$

where *N* is the number of experimental data; *m* is the number of adjusted parameters; *x*_calc_ is the calculated density value according to the modified Tamman–Tait eq (eq 2), and *x*_exp_ is the experimental density value.

### 2.5 Calculation of Density-Derived Properties

Once the density values are achieved, molar volumes for any mixture of C components can be easily calculated, as shown in eq 6.

6 $$V_{m}=\frac{\mathrm{M̅}}{\rho }=\frac{\sum_{i}^{C}x_{i}M_{i}}{\rho }$$

where M̅ is the apparent molar mass and it can be expressed as a function of the molar mass, *M*_*i*_ and the molar fraction, *x*_*i*_ of the components of the system. Molar volumes at every pressure and temperature were calculated. Dependence of *V*_m_ with *p* can be expressed as seen in eq 7 (with calculation steps demonstrated in the Supporting Information).

7 $$V_{m}=V_{m,0}-\kappa_{0}\cdot V_{m,0}\cdot p+\frac{V_{m}^{\prime \prime }}{2}\cdot p^{2}$$

where *V*_m,0_ is the molar volume extrapolated at *p* → 0 and κ_0_ is the isothermal expansion coefficient extrapolated at *p* → 0. *V*_m_^″^ is the second derivative of molar volume with respect to pressure, which is related to the dependence of the isothermal expansion coefficient with pressure. Fitting *V*_m_ vs *p* to quadratic equations allows the calculation of *V*_m,0_ and κ_0_ at each temperature.

### 2.6 Analysis of Multi Elemental Profiles by Inductively Coupled Plasma–Mass Spectrometry

An elemental profile of the metals Cr, Fe, Mo, and Ni was described using an inductively coupled plasma mass spectrometry (ICP–MS) 7800 from Agilent Technologies, for loaded and unloaded aqueous amine solutions after density measurements. The water used to prepare aqueous amine solutions was selected as blank. The metal concentration (expressed in μg/L) helps to assess the densimeter pipeline corrosion effects of the aqueous amine solutions used in the present work when submitted to high pressures (up to 100 MPa) and high temperatures (up to 393.15 K).

## 3 Results and Discussion

### 3.1 Density Measurements of Unloaded Amine Solutions

#### 3.1.1 Density Measurements of Binary Amine Systems

The experimental data obtained for density measurements for binary systems of AP with H_2_O is represented in Table 3. The densities were not measured at 0.1 and 0.5 MPa for the temperatures of 373.15 and 393.15 K since they represent conditions too close to the water boiling point, misleading the density results.

**Table 3:** Experimental Data of the Density (ρ, kg m^–3^) of AP (1) + H_2_O (2) Solutions at Different Mass Concentrations (*w*, wt %), Temperatures (*T*, K), and Pressures (*p*, MPa)^a^ ^,^ ^b^

ρ (kg m^–3^)						
	*T* (K)					
*p* (MPa)	293.15	313.15	333.15	353.15	373.15	393.15
*w*_1_ = 5.11%						
0.1	998.2	991.9	982.5	971.1	n.m.	n.m.
0.5	998.2	992.0	982.7	971.3	n.m.	n.m.
1.0	998.4	992.2	982.9	971.5	958.1	943.2
2.0	998.8	992.6	983.3	971.9	958.6	943.7
5.0	1000.2	993.9	984.6	973.2	960.0	945.1
10.0	1002.3	996.0	986.8	975.4	962.2	947.6
15.0	1004.5	998.0	988.9	977.6	964.5	950.0
20.0	1006.6	1000.1	990.9	979.7	966.7	952.4
30.0	1010.7	1004.2	995.0	983.8	971.1	957.0
40.0	1014.8	1008.1	999.0	988.0	975.3	961.5
50.0	1018.9	1012.0	1002.9	991.9	979.4	965.9
60.0	1022.8	1015.8	1006.7	995.8	983.5	970.1
70.0	1026.7	1019.6	1010.4	999.7	987.5	974.3
80.0	1030.5	1023.3	1014.1	1003.4	991.4	978.4
90.0	1034.2	1026.9	1017.7	1007.0	995.1	982.3
100.0	1037.9	1030.5	1021.2	1010.6	998.8	986.2
*w*_1_ = 10.01%						
0.1	998.9	992.2	982.5	970.9	n.m.	n.m.
0.5	998.9	992.3	982.7	971.1	n.m.	n.m.
1.0	999.1	992.4	982.9	971.3	957.9	942.8
2.0	999.5	992.8	983.3	971.7	958.3	943.3
5.0	1000.8	994.0	984.6	973.0	959.7	944.8
10.0	1002.8	996.1	986.7	975.2	962.0	947.3
15.0	1004.9	998.1	988.7	977.3	964.2	949.7
20.0	1006.9	1000.1	990.8	979.4	966.5	952.1
30.0	1010.9	1004.0	994.7	983.5	970.8	956.7
40.0	1014.8	1007.9	998.6	987.5	975.0	961.1
50.0	1018.7	1011.8	1002.5	991.5	979.1	965.5
60.0	1022.5	1015.4	1006.2	995.3	983.1	969.8
70.0	1026.2	1019.2	1009.9	999.1	987.0	973.9
80.0	1029.9	1022.8	1013.4	1002.7	990.9	977.9
90.0	1033.4	1026.2	1017.0	1006.4	994.6	981.9
100.0	1036.9	1029.6	1020.4	1009.8	998.3	985.7
*w*_1_ = 19.99%						
0.1	1001.5	993.5	982.7	970.3	n.m.	n.m.
0.5	1001.6	993.6	982.9	970.5	n.m.	n.m.
1.0	1001.7	993.7	983.1	970.6	956.5	940.8
2.0	1002.1	994.1	983.5	971.1	956.9	941.3
5.0	1003.3	995.2	984.7	972.4	958.3	942.8
10.0	1005.2	997.2	986.8	974.5	960.5	945.3
15.0	1007.1	999.1	988.7	976.5	962.7	947.6
20.0	1008.9	1001.0	990.7	978.6	964.9	950.0
30.0	1012.6	1004.7	994.5	982.6	969.2	954.6
40.0	1016.2	1008.3	998.3	986.5	973.4	959.0
50.0	1019.8	1011.9	1002.0	990.3	977.1	963.3
60.0	1023.3	1015.4	1005.5	994.0	981.4	967.5
70.0	1026.8	1018.9	1009.1	997.8	985.2	971.6
80.0	1030.2	1022.3	1012.5	1001.4	989.0	975.6
90.0	1033.5	1025.6	1016.0	1004.9	992.6	979.5
100.0	1036.8	1029.0	1019.3	1008.3	996.2	983.3
*w*_1_ = 29.99%						
0.1	1005.4	995.7	983.7	970.4	n.m.	n.m.
0.5	1005.4	995.8	983.9	970.5	n.m.	n.m.
1.0	1005.6	995.9	984.1	970.7	955.9	939.7
2.0	1005.9	996.3	984.4	971.1	956.3	940.2
5.0	1007.0	997.4	985.6	972.4	957.7	941.7
10.0	1008.8	999.2	987.6	974.5	959.9	944.2
15.0	1010.5	1001.0	989.6	976.5	962.1	946.6
20.0	1012.3	1002.8	991.4	978.6	964.3	949.0
30.0	1015.7	1006.3	995.1	982.5	968.5	953.5
40.0	1019.0	1009.8	998.8	986.3	972.6	957.9
50.0	1022.3	1013.2	1002.3	990.0	976.6	962.2
60.0	1025.6	1016.6	1005.8	993.7	980.5	966.4
70.0	1028.8	1019.8	1009.2	997.3	984.2	970.5
80.0	1031.9	1023.1	1012.5	1000.8	988.0	974.4
90.0	1035.0	1026.2	1015.8	1004.2	991.6	978.2
100.0	1037.9	1029.3	1018.9	1007.5	995.0	982.0
*w*_1_ = 39.99%						
0.1	1010.1	998.6	985.4	971.1	n.m.	n.m.
0.5	1010.1	998.7	985.5	971.1	n.m.	n.m.
1.0	1010.3	998.8	985.7	971.4	955.7	938.9
2.0	1010.6	999.1	986.1	971.8	956.1	939.3
5.0	1011.6	1000.2	987.2	973.0	957.5	940.9
10.0	1013.4	1002.0	989.2	975.1	959.7	943.3
15.0	1015.0	1003.7	991.1	977.1	961.9	945.7
20.0	1016.6	1005.5	992.9	979.2	964.1	948.1
30.0	1019.9	1008.9	996.5	983.0	968.4	952.7
40.0	1023.0	1012.2	1000.1	986.9	972.4	957.2
50.0	1026.2	1015.5	1003.6	990.6	976.4	961.5
60.0	1029.3	1018.8	1007.0	994.2	980.3	965.6
70.0	1032.3	1022.0	1010.3	997.7	984.0	969.6
80.0	1035.3	1025.1	1013.6	1001.2	987.7	973.6
90.0	1038.2	1028.1	1016.8	1004.5	991.4	977.4
100.0	1041.1	1031.1	1019.9	1007.8	994.8	981.2

a Expanded uncertainties (*k* = 2) are *U*(*T*) = 0.02 K; *U*_r_(*p*) = 0.0002; *U*(*w*) = 0.0002; and *U*(ρ) = 0.7 kg·m^–3^.

b n.m. = not measured.

To our knowledge, there are no density data for system AP + H_2_O at high pressure. There are, however, some references for data at atmospheric pressure: Hartono and Knuutila^18^ determined densities for the whole concentration range ant temperatures between 293.15 and 363.15 K. Data from Islam et al.^19^ were also determined for the whole composition range and between 298.15 and 323.15 K. Comparison of data in this work at 0.1 MPa with literature was performed. In the present work, molar fractions ranging between 0.0128 and 0.1378 (corresponding to percent mass fractions of 5.11% to 39.99%, respectively) were determined. The results agree very well with literature data (Figure S3). Average absolute deviations (AAD) between experimental data and Hartono and Knuutila^18^ at 293.15, 313.15, and 333.15 K are 0.4, 0.4, and 0.7 kg·m^–3^ respectively, while AAD between our data and Islam et al.^19^ at 313.15 K is 0.6 kg·m^–3^.

In comparison to a recent work reported by Hartono and Knuutila,^18^ who also analyzed AP + H_2_O across different concentrations and temperature ranges, the lowest AP concentrations under ambient pressure yielded densities similar to those observed in this study. In the present work, molar fractions of 0.0128–0.1378 (corresponding to mass fractions of 5.11–39.99%, respectively) resulted in densities of 998.2–1010.1 kg·m^–3^ at 293.15 K, whereas by interpolation of data by Hartono and Knuutila^18^ a density range of 998.8–1010.1 kg·m^–3^ is obtained for the same concentration range. At 353.15 K, small differences were also observed, with densities of 971.1–971.1 kg·m^–3^ in this study compared to 971.8–971.4 kg·m^–3^ in their work.

Density increases with pressure and decreases with temperature as expected, but in order to visualize more insightful trends, derived properties must be calculated. Figure 2 demonstrates the values of *V*_m,0_ and κ_0_ vs *T* for the system AP + H_2_O at every composition measured. It also includes the values for pure water calculated using REFPROP software.^20^

**Figure 2:**
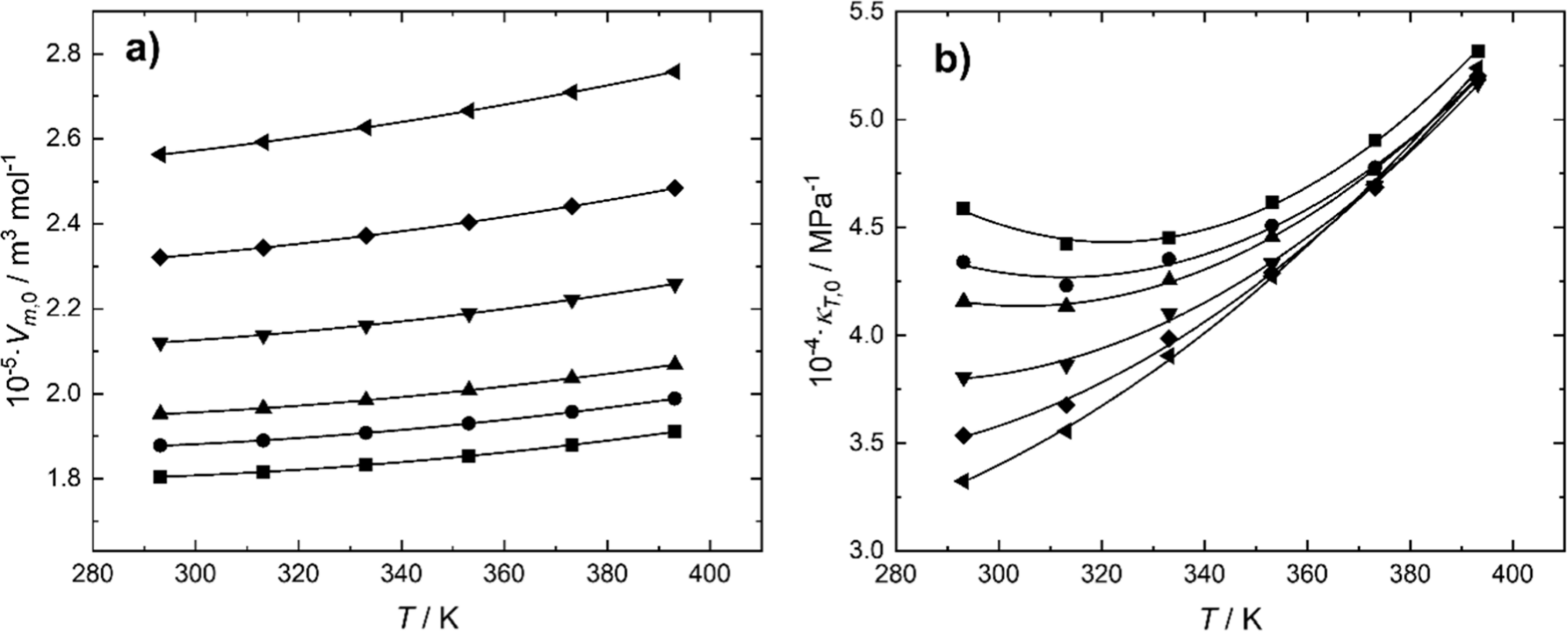
(a) Molar volume (b) isothermal expansion coefficient, both extrapolated at zero pressure for the system AP (1) + H_2_O (2) at various AP concentrations. (■): *w*_1_ = 0%. (●): *w*_1_ = 5.11%. (▲): *w*_1_ = 10.01%. (▼): *w*_1_ = 19.99%. (◆): *w*_1_ = 29.99%. (◀): *w*_1_ = 39.99%.

The molar volume increases with temperature as expected and increases with AP concentration, also expected as the molar volume for the amine is significantly higher than for H_2_O. If *V*_m,0_ is plotted against *x*_AP_ (Figure S1, Supporting Information), a linear trend is observed. That can only happen if the excess molar volume is close to zero or if it follows a linear tendency with concentration withing the composition range studied. Excess molar volumes for AP + H_2_O systems were previously measured and resulted in negative values over the whole range of compositions^18,19^ and the values were significant, reaching a minimum of −0.9 cm^3^·mol^–1^ at 298.15 K. In this particular case, *V*_m_^*E*^ vs *x*_AP_ follows a tendency almost linear for AP mole fractions lower than 0.14. Therefore, this could be a reasonable explanation for the linear trend of *V*_m_ vs *x*_AP_ observed in this work.

The isothermal expansion coefficient generally increases with the temperature. However, at low concentrations of AP, a local minimum is observed. This behavior is likely due to the contribution of the anomalous behavior of isothermal compressibility of water, which decreases with temperature up to a minimum at 319 K^21^ as also shown in Figure 2. Moreover, it is noticed that the isothermal compressibility decreases with the concentration of amine, particularly at low temperatures. That may be due to attractive intermolecular interactions, which are plausible with negative molar volumes.

#### 3.1.2 Density Measurements of Tertiary Amine Systems

The experimental data obtained for density measurements for tertiary systems of AP, AMP, and water are represented in Table 4. Similar to binary systems, the densities were not measured at 0.1 and 0.5 MPa for the temperatures of 373.15 and 393.15 K.

**Table 4:** Experimental Data of the Density (ρ, kg m^–3^) of AP (1) + AMP (2) + H_2_O (3) Solutions at Different Mass Concentrations (*w*, wt %), Temperatures (*T*, K), and Pressures (*p*, MPa)^a^ ^,^ ^b^

ρ (kg m^–3^)						
	*T* (K)					
*p* (MPa)	293.15	313.15	333.15	353.15	373.15	393.15
AP/AMP (1:2): *w*_1_ = 10.00%; *w*_2_ = 19.98%						
0.1	1000.1	995.6	989.7	982.8	n.m.	n.m.
0.5	1000.1	995.6	989.7	983.0	n.m.	n.m.
1.0	1000.2	995.8	989.7	983.0	974.1	964.0
2.0	1000.6	996.1	990.4	983.5	974.9	964.8
5.0	1001.9	997.2	991.4	984.8	976.2	966.1
10.0	1003.6	999.1	993.6	987.0	978.5	969.0
15.0	1005.3	1000.9	995.6	989.3	980.7	971.3
20.0	1006.9	1002.8	997.5	991.1	983.2	974.3
30.0	1010.4	1006.3	1001.4	995.4	987.6	979.2
40.0	1013.7	1009.7	1005.1	999.5	991.8	983.8
50.0	1017.0	1013.4	1008.6	1003.4	996.2	988.1
60.0	1020.2	1016.7	1012.2	1007.1	1000.2	992.6
70.0	1023.4	1020.0	1015.7	1010.8	1004.2	997.0
80.0	1026.5	1023.2	1019.3	1014.5	1008.2	1001.5
90.0	1029.6	1026.4	1022.6	1018.1	1011.7	1005.1
100.0	1032.6	1029.5	1025.9	1021.6	1015.6	1008.7
AP/AMP (1:1): *w*_1_ = 15.00%; *w*_2_ = 15.01%						
0.1	1002.6	992.2	979.4	965.1	n.m.	n.m.
0.5	1002.6	992.2	979.6	965.3	n.m.	n.m.
1.0	1002.8	992.3	979.8	965.5	949.8	933.0
2.0	1003.1	992.7	980.2	965.9	950.3	933.6
5.0	1004.2	993.8	981.4	967.2	951.7	935.1
10.0	1005.9	995.6	983.4	969.4	953.9	937.6
15.0	1007.7	997.5	985.3	971.4	956.2	940.1
20.0	1009.4	999.3	987.3	973.5	958.4	942.6
30.0	1012.8	1002.8	991.1	977.5	962.9	947.3
40.0	1016.1	1006.3	994.7	981.4	967.1	951.8
50.0	1019.4	1009.8	998.3	985.2	971.1	956.2
60.0	1022.7	1013.1	1001.7	988.9	975.1	960.6
70.0	1025.9	1016.4	1005.2	992.5	978.9	964.7
80.0	1029.0	1019.6	1008.5	996.0	982.7	968.6
90.0	1032.1	1022.7	1011.7	999.5	986.3	972.6
100.0	1035.1	1025.8	1014.9	1002.9	990.0	976.4
AP/AMP (2:1): *w*_1_ = 20.01%; *w*_2_ = 10.02%						
0.1	1003.6	993.4	981.0	967.2	n.m.	n.m.
0.5	1003.5	993.4	981.2	967.4	n.m.	n.m.
1.0	1003.7	993.6	981.3	967.5	952.3	935.6
2.0	1004.0	993.9	981.8	968.0	952.8	936.1
5.0	1005.2	995.1	983.0	969.3	954.2	937.7
10.0	1006.9	996.9	985.0	971.4	956.4	940.2
15.0	1008.7	998.7	986.9	973.5	958.7	942.6
20.0	1010.4	1000.6	988.8	975.5	960.8	945.1
30.0	1013.8	1004.1	992.5	979.5	965.2	949.7
40.0	1017.1	1007.5	996.2	983.4	969.4	954.2
50.0	1020.5	1010.9	999.8	987.2	973.4	958.6
60.0	1023.7	1014.3	1003.3	990.9	977.3	962.8
70.0	1026.9	1017.6	1006.7	994.5	981.1	966.9
80.0	1030.0	1020.9	1010.2	998.1	984.9	970.9
90.0	1033.1	1024.0	1013.4	1001.5	988.6	974.8
100.0	1036.2	1027.2	1016.6	1004.8	992.1	978.6

a Expanded uncertainties (*k* = 2) are *U*(*T*) = 0.02 K; *U*_r_(*p*) = 0.0002; *U*(*w*) = 0.0002; and *U*(ρ) = 0.7 kg·m^–3^.

b n.m. = not measured.

Varying the proportion of AP and AMP does not have much effect on both the molar volume and the isothermal compressibility (Figure S2, Supporting Information). In this case, all the amine solutions behave similarly, particularly at low temperature. For example, at 298.15 K, the molar volumes, *V*_m,0_, range between 2.34 × 10^–5^ and 2.36 × 10^–5^ kg m^–3^ and the isothermal compressibility κ_0_, between 3.56 × 10^–4^ and 3.59 × 10^–4^ MPa^–1^.

### 3.2 Density Measurements of CO_2_-Loaded Amine Solutions

The AP aqueous solution at 30 wt % was loaded with CO_2_ as described in Section 2.3.2. The amine concentration was fixed at 30.01 wt %, and the pH of the same solution was 13.3 before CO_2_-loading and 8.2 after achieving CO_2_ saturation. This solution works as a stock solution for the preparation of different CO_2_-loadings (α, mol CO_2_ mol amine^–1^) of 0.2, 0.4, and 0.6, where the results for experimental density measurements are presented in Table 5.

**Table 5:** Experimental Data of the Density (ρ, kg m^–3^) of CO_2_-Loaded Aqueous AP (*w* = 30.01 wt %) Solutions at Different CO_2_ Loadings (α, mol CO_2_ mol amine^–1^), Temperatures (*T*, K), and Pressures (*p*, MPa)^a^ ^,^ ^b^

ρ (kg m^–3^)						
	*T* (K)					
*p* (MPa)	293.15	313.15	333.15	353.15	373.15	393.15
α = 0.194						
0.5	1040.1	1026.1	1014.2	1001.6	n.m.	n.m.
1.0	1040.2	1026.0	1014.4	1001.7	n.m.	n.m.
2.0	1040.3	1026.1	1014.8	1002.2	988.1	972.9
5.0	1041.2	1027.1	1015.9	1003.4	989.5	974.3
10.0	1042.9	1028.9	1017.8	1005.3	991.5	976.6
15.0	1044.6	1030.6	1019.7	1007.3	993.6	978.8
20.0	1046.3	1032.4	1021.5	1009.2	995.7	981.1
30.0	1049.6	1035.8	1025.1	1013.0	999.7	985.4
40.0	1052.8	1039.1	1028.6	1016.7	1003.6	989.6
50.0	1056.1	1042.5	1032.1	1020.3	1008.4	994.6
60.0	1059.3	1045.8	1035.4	1023.8	1012.2	998.6
70.0	1062.4	1049.0	1038.8	1027.3	1015.8	1002.5
80.0	1065.5	1052.2	1042.0	1030.7	1018.4	1005.2
90.0	1068.5	1055.2	1045.3	1034.0	1021.8	1008.9
100.0	1071.5	1058.3	1048.4	1037.3	1025.2	1012.5
α = 0.408						
0.5	1066.3	1052.9	1041.9	1029.8	n.m.	n.m.
1.0	1066.2	1053.0	1042.1	1029.9	n.m.	n.m.
2.0	1065.0	1053.3	1042.5	1030.3	1016.8	1002.3
5.0	1066.0	1054.4	1043.6	1031.5	1018.1	1003.6
10.0	1067.7	1056.1	1045.5	1033.4	1020.1	1005.6
15.0	1069.4	1057.9	1047.3	1035.3	1022.0	1007.8
20.0	1071.1	1059.5	1049.1	1037.2	1024.0	1009.9
30.0	1074.3	1063.0	1052.6	1040.8	1027.9	1014.0
40.0	1077.5	1066.3	1056.0	1044.4	1031.7	1018.2
50.0	1080.8	1069.6	1059.4	1047.9	1035.4	1022.1
60.0	1083.9	1072.7	1062.7	1051.3	1039.0	1025.9
70.0	1087.0	1076.0	1066.0	1054.7	1042.3	1029.5
80.0	1090.1	1079.1	1069.2	1058.0	1045.9	1033.0
90.0	1093.0	1082.2	1072.3	1061.2	1049.3	1036.6
100.0	1096.0	1085.2	1075.4	1064.4	1052.6	1040.1
α = 0.611						
0.5	1087.6	1077.8	1066.9	1054.8	n.m.	n.m.
1.0	1087.5	1077.9	1067.0	1054.9	n.m.	n.m.
2.0	1087.7	1078.3	1067.4	1055.3	1042.0	1027.4
5.0	1088.8	1079.3	1068.5	1056.5	1043.2	1028.7
10.0	1090.5	1081.0	1070.3	1058.3	1045.1	1030.8
15.0	1092.1	1082.8	1072.1	1060.2	1047.1	1032.9
20.0	1093.8	1084.5	1073.8	1062.0	1049.0	1034.9
30.0	1097.1	1087.9	1077.2	1065.6	1052.8	1039.0
40.0	1100.3	1091.1	1080.7	1069.0	1056.4	1042.9
50.0	1103.5	1094.4	1084.0	1072.5	1060.0	1046.7
60.0	1106.7	1097.6	1087.2	1075.8	1063.6	1050.4
70.0	1109.8	1100.7	1090.4	1079.2	1067.0	1054.0
80.0	1112.9	1103.9	1093.6	1082.4	1070.4	1057.6
90.0	1115.9	1106.9	1096.7	1085.5	1073.7	1061.0
100.0	1118.9	1109.9	1099.7	1088.7	1076.9	1064.5

a Expanded uncertainties (*k* = 2) are *U*(*T*) = 0.02 K; *U*_r_(*p*) = 0.0002; *U*(*w*) = 0.0002; *U*_r_(α) = 0.03 and *U*(ρ) = 1.8 kg·m^–3^.

b n.m. not measured.

It is important to notice that no experiments at 0.1 MPa were conducted due to the CO_2_ solubility limits in aqueous amine solutions. According to Dong et al.,^22^ the pressure limit for α = 0.510 in an AP solution (*M* = 4.0 mol·dm^–3^, which is approximately 30 wt %) at 393.15 K is 0.4288 MPa. Therefore, only pressures higher than 0.5 MPa were employed for temperatures under 373.15 K and pressures higher than 1.0 MPa were applied for 373.15 and 393.15 K to ensure all CO_2_ would be solubilized in the aqueous amine solution during all of the density measurement.

Figure 3 presents the curves of *V*_m,0_ and κ_0_ vs *T* for the system AP + H_2_O + CO_2_ at the constant concentration of AP = 30% and increasing CO_2_ loadings.

**Figure 3:**
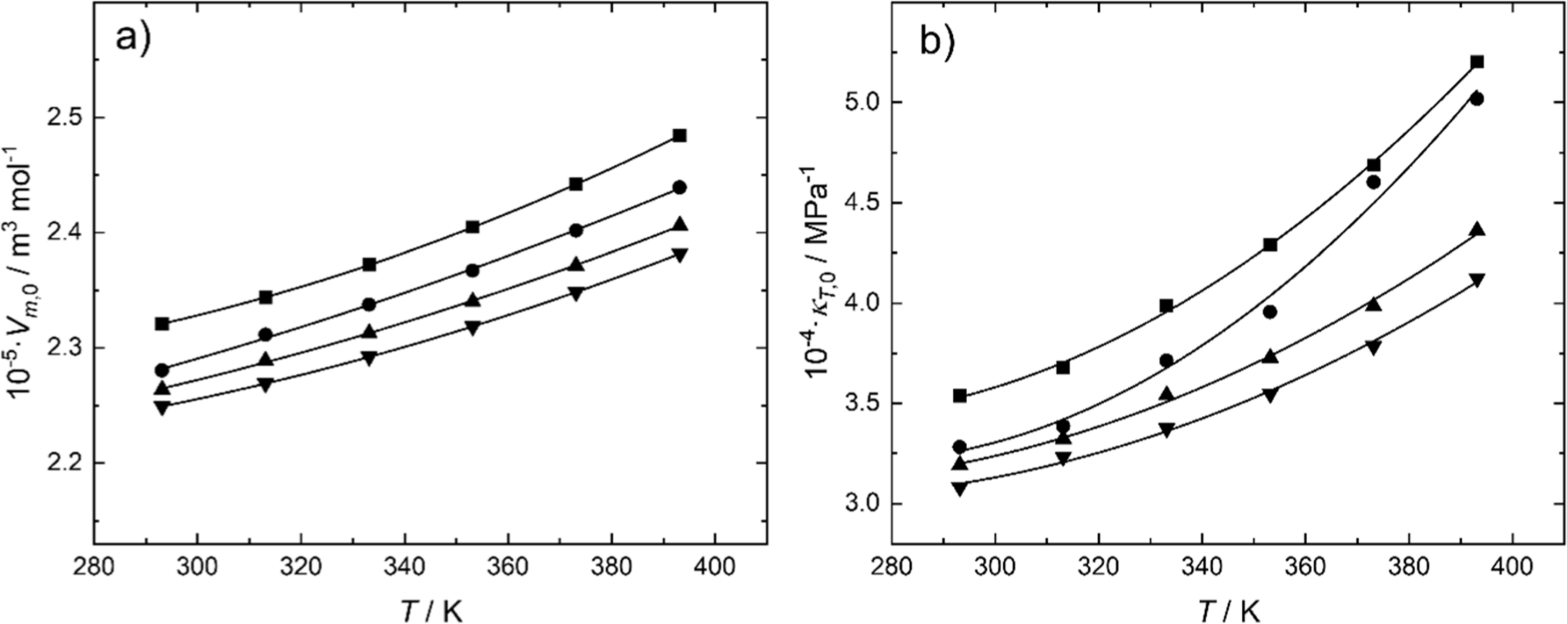
(a) Molar volume (b) isothermal expansion coefficient, both extrapolated at zero pressure for the system AP (1) + H_2_O (2), *w*_1_ = 30.0% with CO_2_ at various loadings. (■): α = 0. (●): α = 0.194. (▲): α = 0.408. (▼): α = 0.611.

The molar volume decreases with the loading of CO_2_, as does the isothermal expansion coefficient. Dissolution of CO_2_ in pure water is very low, due to the high energy required to break the water H-bond network but for mixtures H_2_O + amine, the solubility increases significantly because amine reacts with carbon dioxide to form carbamates: 2R-NH_2_ + CO_2_ ⇆ [R-NH-COO^–^]­[R-NH_3_^+^]. In this reaction, CO_2_ and the amine form an adduct (carbamic acid), which reacts with another amine to form the carbamate of the amine, an ionic species. The contraction of the volume upon CO_2_ addition can be explained by electrostriction of the solvent caused by the presence of electric fields generated by the ionic species.^23^ Hawrylak et al.^24^ studied an analogous situation. They measured densities for methyldiethanolamine (MDEA) at the counterpart chloride methyldiethanolamonium (MDEAH^+^Cl^–^). Using their data, molar partial volumes for water were calculated at 298 K. They decreased with the concentration of the solute for both the amine and chloride, but the effect of the latter was more pronounced. Rough calculations of the isothermal compressibilities were done and they resulted significantly lower for MDEAH^+^Cl^–^ than for MDEA solutions.

### 3.3 Tammann–Tait Density Correlation

Density isotherms for the binary, tertiary, and CO_2_-loaded systems are presented in Figures 4a–e, 5a–c, and 6a–c, where the behavior of the density as a function of pressure is observed for the amine solutions at different concentrations. In addition, the modified Tammann–Tait fitting is also depicted in Figures 4–6. The value of fitting parameters and statistical analysis (eqs 2–5) to validate the quality of the model were calculated for each amine concentration and can be seen in Table 6.

**Figure 4:**
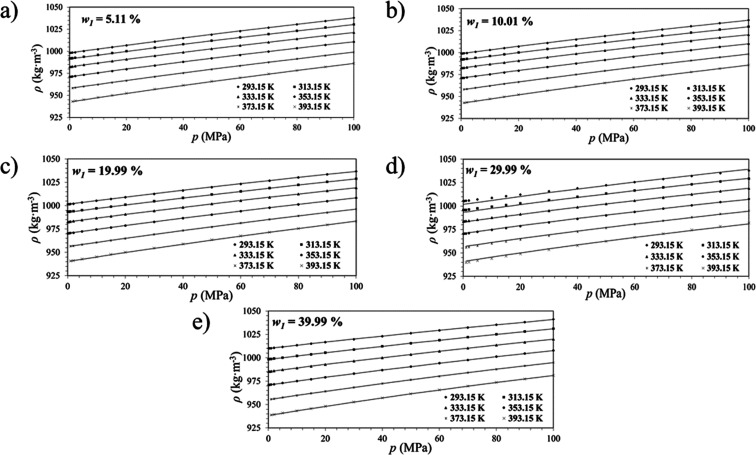
Densities isotherms for AP (1) + H_2_O (2) solutions as a function of pressure and their respective model fittings (eq 2). The AP mass percentages are (a) *w*_1_ = 5.11%; (b) *w*_1_ = 10.01%; (c) *w*_1_ = 19.99%; (d) *w*_1_ = 29.99%, and e) *w*_1_ = 39.99%.

**Figure 5:**
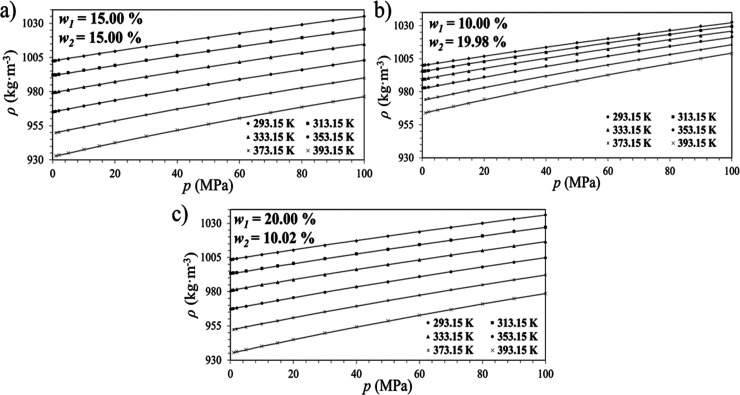
Densities isotherms for AP (1) + AMP (2) + H_2_O (3) solutions as a function of pressure and their respective model fittings (eq 2). The AP/AMP mass proportions are (a) 1:1; (b) 1:2, and (c) 2:1. The exact mass concentration (*w*_*i*_) of each amine is highlighted in each graph.

**Figure 6:**
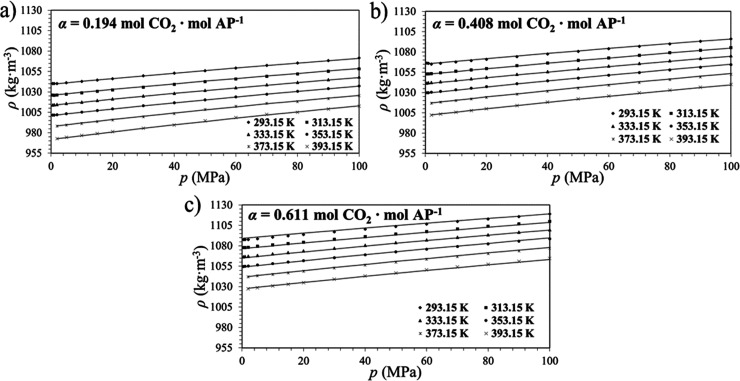
Densities isotherms for CO_2_-loaded aqueous AP solutions (*w* = 30.01 wt %) as a function of pressure and their respective model fittings (eq 2). The CO_2_-loadings (α, mol CO_2_ mol amine^–1^) are (a) 0.194, (b) 0.408, and (c) 0.611.

**Table 6:** Modified Tammann–Tait (Equation 2) Fitting Parameters and Statistical Analysis of the Modeling Applied for Experimental Density Data Measured for Binary, Tertiary, and CO_2_-Loaded Amine Systems

AP (1) + H_2_O (2)					
*w*_1_ (wt %)	5.11	10.01	19.99	29.99	39.99
*p*_ref_ (MPa)	1.0	1.0	1.0	1.0	1.0
*A*_0_ (kg m^–3^)	853.593	870.809	911.283	911.283	1030.37
*A*_1_ (kg m^–3^ K^–1^)	1.27924	1.18768	0.99670	0.99670	0.41421
*A*_2_ (kg m^–3^ K^–2^)	–0.00268	–0.00256	–0.00235	–0.00235	–0.00165
*B*_0_ (MPa)	–579.727	–396.597	–91.214	–569.259	575.207
*B*_1_ (MPa K^–1^)	5.5871	4.5624	3.0777	4.8785	–0.62809
*B*_2_ (MPa K^–2^)	–0.00884	–0.00750	–0.00570	–0.00755	–0.00079
*C*	0.13249	0.12633	0.12558	0.09348	0.11044
σ (kg m^–3^)	0.21	0.19	0.17	1.29	0.09
MD (%)	0.05	0.05	0.04	0.36	0.03
AAD (%)	0.02	0.02	0.01	0.09	0.01
AP (1) + AMP (2) + H_2_O (3)					
*w*_1_; *w*_2_ (wt %)	10.00; 19.98	15.00; 15.00	20.00; 10.02		
*p*_ref_ (MPa)	1.0	1.0	1.0		
*A*_0_ (kg m^–3^)	902.550	979.684	974.578		
*A*_1_ (kg m^–3^ K^–1^)	0.85095	0.66127	0.68360		
*A*_2_ (kg m–3 K^–2^)	–0.00177	–0.00199	–0.00199		
*B*_0_ (MPa)	471.745	326.009	362.549		
*B*_1_ (MPa K^–1^)	–0.3627	0.6354	0.4878		
*B*_2_ (MPa K^–2^)	–0.00094	–0.00246	–0.00224		
*C*	0.10428	0.10938	0.11349		
σ (kg m^–3^)	0.22	0.18	0.12		
MD (%)	0.05	0.04	0.03		
AAD (%)	0.02	0.02	0.01		
CO_2_-Loaded AP (1) + H_2_O (2); *w*_1_ = 30.01 wt %					
α (mol CO_2_ mol amine^–1^)	0.194	0.408	0.611		
*p*_ref_ (MPa)	2.0	2.0	2.0		
*A*_0_ (kg m^–3^)	2108.077	2131.230	2153.889		
*A*_1_ (kg m^–3^ K^–1^)	–8.63599	–8.63594	–8.63588		
*A*_2_ (kg m^–3^ K^–2^)	0.02410	0.02410	0.02410		
*B*_0_ (MPa)	339.788	1188.975	1818.640		
*B*_1_ (MPa K^–1^)	0.5282	–4.1131	–7.8908		
*B*_2_ (MPa K^–2^)	–0.00216	0.00458	0.01018		
*C*	0.10570	0.11913	0.11473		
σ (kg m^–3^)	0.43	0.57	1.02		
MD (%)	0.08	0.10	0.19		
AAD (%)	0.03	0.05	0.08		

The fitted curves confirmed excellent agreement with experimental data, capturing the nonlinear compressibility behavior of the solutions at high pressures. Moreover, when the solvent density influences the contact between gas and liquid phases, this kind of correlation contributes directly to the optimization of gas absorption systems.

### 3.4 Assessing the Effects of Amine Composition and Dissolved CO_2_ in Metal Corrosion

As density of the amines’ solution plays an important role in the CO_2_ capture process, corrosion of the carbon steel is also an important parameter in process design. For instance, it was estimated that costs of corrosion could represent around 6% of the total Gross National Products (GNP) in the United States, i.e., over half billion dollars had to be spend in covering corrosion costs.^7^ The influence on the metal corrosion of the amine concentration, composition, and CO_2_ loaded amount (α) in the aqueous amine solutions is presented in terms of elemental analysis (Figure 7).

**Figure 7:**
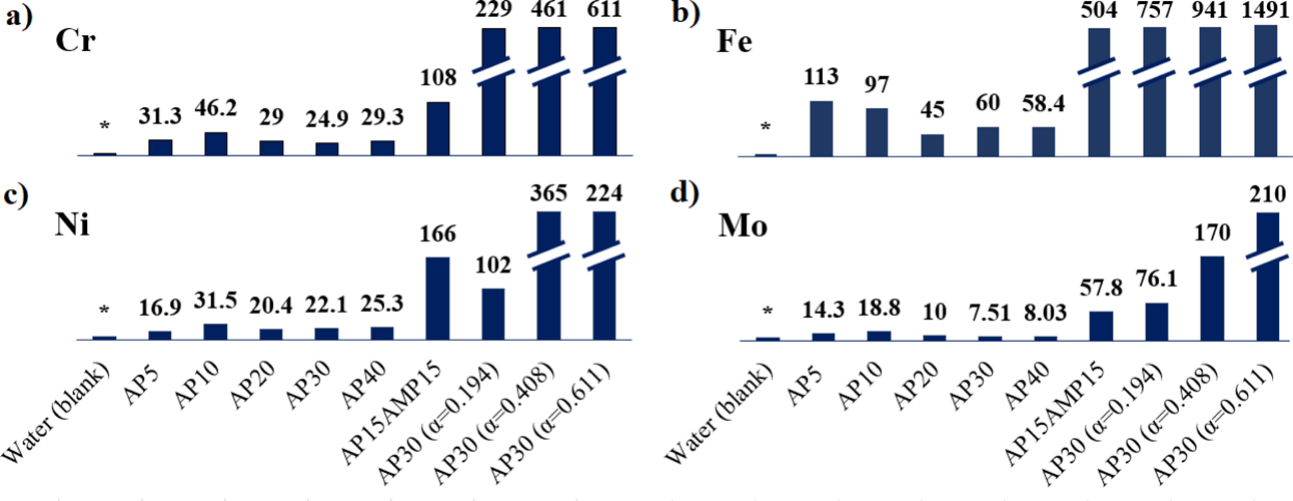
Elemental analysis of different aqueous amine solutions after density measurements. Elemental analysis in terms of metal concentration (μg/mL) of (a) Cr, (b) Fe, (c) Ni and (d) Mo. AP refers to 3-amino-1-propanol while AMP represents 2-amino-2-methyl-1-propanol. The number in front of the amine abbreviation refers to the percentage in the aqueous amine solution. α is the molar concentration of CO_2_ of loaded amines (mol CO_2_/mol AP). *Metal concentration ≤5 μg/mL according to the specification sheet of pure water (CAS 7732-18-5).

Alkanolamine solutions themselves are usually not corrosive,^25^ and the degree of corrosiveness will rely on the intrinsic characteristics of oxidants and corrosion products that are present in the process performed near ambient pressures.^26^ However, under high pressure conditions, limited information is known about its corrosiveness effects. Figure 7 demonstrates, after metal analysis of solutions submitted to ultra high-pressure conditions, the concentration of the metals presented in the solution, where Cr, Fe, Ni, and Mo were selected to predict the level of corrosiveness effect. As observed, a certain degree of corrosion has happened to all solutions (compared to the metal concentration in the blank, water in this case). AP solutions appear to be little corrosive, regardless of the concentration. Amines are known to exert metal corrosiveness when they absorb CO_2_, which is a primary corrosion agent,^27^ particularly at high temperatures, corroborating the clearly higher values found for all the four metals analyzed. Alkanolamine solutions can degrade in the presence of CO_2_ and O_2_, the decomposition products being potential corrosive agents to the steel, a limitation to bear in mind for material selection when designing reactors. Interestingly, in the presence of AMP, the concentration of the metals in solution increases, turning to be even higher than loaded amine solutions in the case of Ni. In fact, comparative studies of corrosion by different loaded amine solutions conclude that AMP is in general one of the most corrosive alkylamines,^28^ which corroborates the result of tertiary amine solution results in this work.

## 4 Conclusion

Comprehensive thermophysical data for aqueous CO_2_-loaded AP, unloaded-AP, and unloaded AP + AMP aqueous systems are reported in this work. More specifically, density was determined experimentally, while molar volume and isothermal expansion coefficient were successfully calculated. These data are of great relevance in CO_2_ capture technologies. The experimental results confirmed that solution density increases with pressure and decreases with temperature and that the addition of CO_2_ results in significant changes in molar volume and expansion behavior. In addition, the modified Tammann–Tait equation presented excellent correlation with density data. Notably, the presence of AMP and higher CO_2_ loadings worsen corrosivity, reinforcing the need for cautious material selection when working with these systems and considering the incorporation of inhibitors, especially under high-pressure conditions. Finally, the reported properties presented in this work provide valuable data for future accurate process modeling, equipment design, and optimization of CO_2_ absorption systems using aqueous alkanolamine solvents.

## Supplementary Material



## Notes

Representation of *V*_m,0_ vs *x*_AP_ for the system H_2_O + AP at 293.15 and 333.15 K; molar volume and isothermal compressibility at the limit of zero pressure and 298.15 K for the system H_2_O + AP + AMP at different mole fractions of AP; the mole fraction of water is kept almost constant (ranging from 0.911 to 0.916); and comparison between data in this work (●) and literature data at 293 K, 313.15 K, and 333.15 K; (×): Hartono and Knuutila,^18^ (◯): Islam et al.^19^ (PDF)
