# Supplementary material for: Densities of CO2‑Loaded and Unloaded 3‑Amino-1-propanol Aqueous Solutions and Their Blends with 2‑Amino-2-methyl-1-propanol at High Pressures
Source: ACS Omega. 2025 Oct 24;10(43):51419–30. doi: 10.1021/acsomega.5c07053 (PMC12593155; doi:10.1021/acsomega.5c07053)
Supplement: Supplementary file 1 [file ao5c07053_si_001.pdf]

Densities of CO_2 loaded and unloaded 3-Amino-1-propanol aqueous solutions and their blends with 2-Amino-2-methyl-1-propanol at high pressures.

Luana C. dos Santos[1,*], Eduardo Pérez[2], Alejandro Moreau[3], María D. Bermejo[4], Jose J. Segovia[3]

[1] Foodomics Laboratory, Instituto de Investigación en Ciencias de la Alimentación (CIAL, CSIC-UAM), Nicolás Cabrera 9, Campus de Cantoblanco, 28049 Madrid, Spain

[2] Physical Chemistry Department, Universidad Complutense de Madrid, Madrid, 28040, Spain

[3] BioEcoUva Research Institute on Bioeconomy, TERMOCAL-Thermodynamics and Calibration, University of Valladolid, Valladolid, 47011, Spain

[4] BioEcoUva Research Institute on Bioeconomy, PressTech, Department of Chemical Engineering and Environmental Technology, Universidad de Valladolid, Valladolid 47011, Spain

**corresponding author:* luana.dsantos@csic.es

Supplementary information

Figure S1 shows the molar volume extrapolated at P = 0 against AP mole fraction at 293.15 and 333.15 K for the system H_2O + AP. A linear trend is observed. That can only happen if the excess molar volume is close to zero or if it follows a linear tendency with concentration withing the composition range studied.

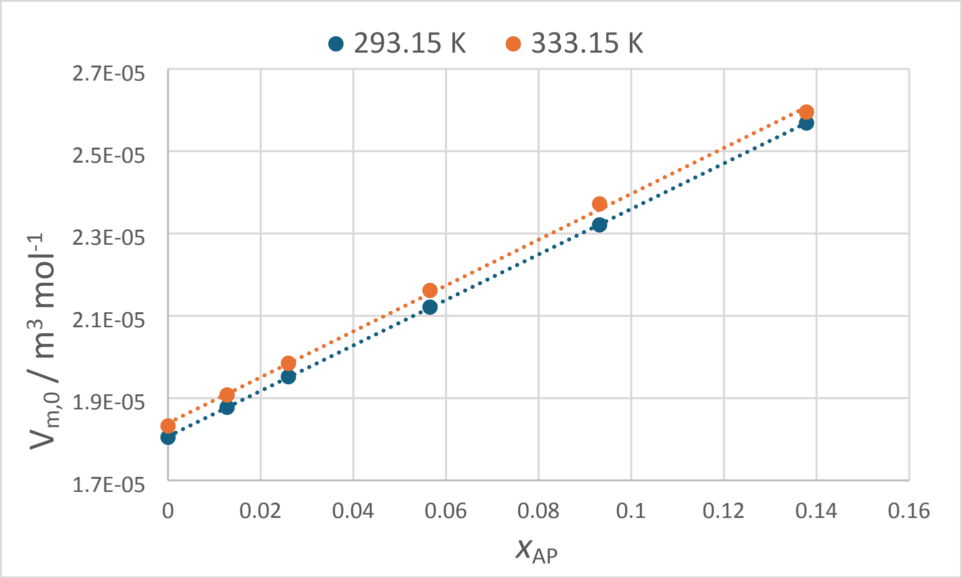

Figure S1. Representation of $V_{m,0}$ vs x_{AP} for the system for the system H_2O + AP at 293.15 and 333.15 K

Varying the proportion of AP and AMP does not have much effect on both the molar volume and the isothermal compressibility (Figure S2).

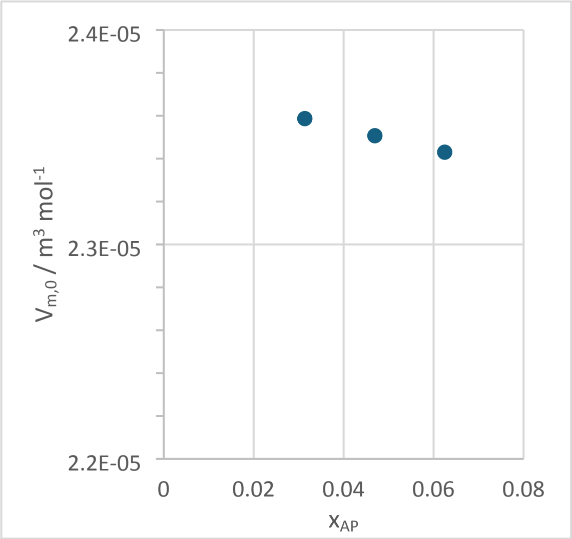

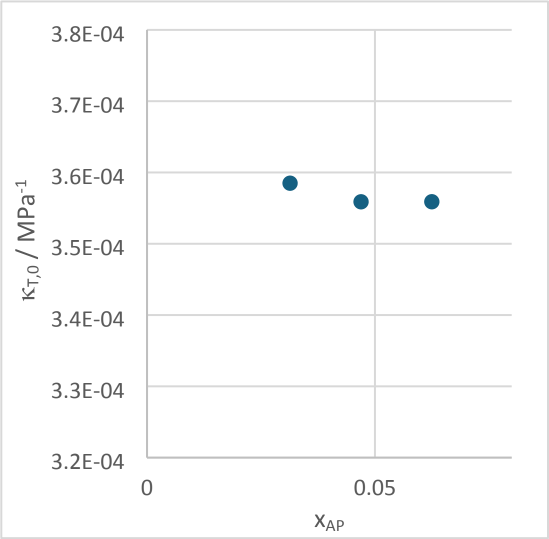

Figure S2. Molar Volume (left) and Isothermal compressibility (right) at the limit of zero pressure and 298.15 K for the system H_2O + AP + AMP at different mole fraction of AP. The mole fraction of water is kept almost constant (ranging from 0.911 to 0.916).

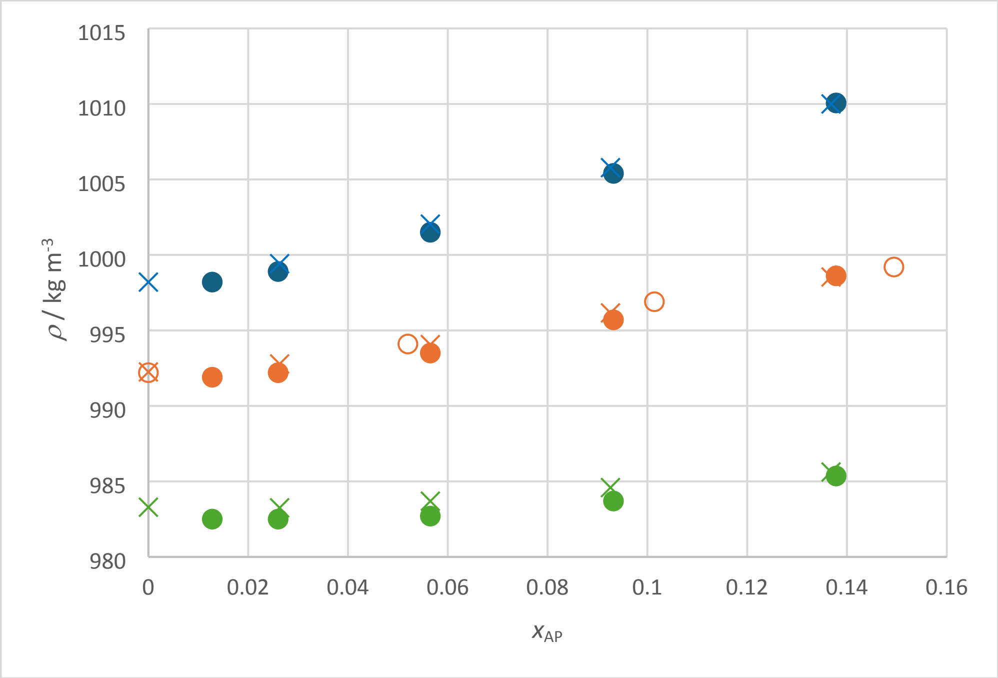

Figure S3. Comparison between data in this work (●) and literature data at 293 K (blue), 313.15 K (orange) and 333.15 K (green). (×): Hartono and Knuutila[18], (○): Islam et al.[19]

Calculation of derived properties

Dependence of V_m with p can be fitted to a quadratic expression to whose coefficients can be given a physical meaning. For a fixed temperature:

$$V_m = a + b \cdot P + c \cdot P^2$$

The molar volume at the limit of zero pressure, $V_{m,0} = a$

Providing that the Isothermal compressibility is: $\kappa_T = -\frac{1}{V_m}\left(\frac{\partial V_m}{\partial P}\right)_T$, then the isothermal compressibility at the limit of zero pressure is: $\kappa_{T,0} = -\frac{b}{V_{m,0}}$

The coefficient c is related to the second derivative of the molar volume respect pressure:

$$V''_m = \left(\frac{\partial^2 V_m}{\partial P^2}\right)_T = 2c$$

Replacing a, b, c in the quadratic expression of V_m:

$$V_m = V_{m,0} - \kappa_0 \cdot V_{m,0} \cdot p + \frac{V''_m}{2} \cdot p^2$$
